# Endotoxemia by *Porphyromonas gingivalis* Alters Endocrine Functions in Brown Adipose Tissue

**DOI:** 10.3389/fcimb.2020.580577

**Published:** 2021-01-19

**Authors:** Masahiro Hatasa, Yujin Ohsugi, Sayaka Katagiri, Sumiko Yoshida, Hiromi Niimi, Kazuki Morita, Yosuke Tsuchiya, Tsuyoshi Shimohira, Naoki Sasaki, Shogo Maekawa, Takahiko Shiba, Tomomitsu Hirota, Haruka Tohara, Hirokazu Takahashi, Hiroshi Nitta, Takanori Iwata

**Affiliations:** ^1^Department of Periodontology, Graduate School of Medical and Dental Sciences, Tokyo Medical and Dental University (TMDU), Tokyo, Japan; ^2^Oral Diagnosis and General Dentistry, Dental Hospital, Tokyo Medical and Dental University (TMDU), Tokyo, Japan; ^3^Division of Molecular Genetics, Research Center for Medical Sciences, The Jikei University School of Medicine, Tokyo, Japan; ^4^Dysphagia Rehabilitation, Department of Gerontology and Gerodontology, Graduate School of Medical and Dental Sciences, Tokyo Medical and Dental University (TMDU), Tokyo, Japan; ^5^Division of Metabolism and Endocrinology, Facility of Medicine, Saga University, Saga, Japan; ^6^Liver Center, Saga University Hospital, Saga, Japan

**Keywords:** endotoxemia, obesity, *Porphyromonas gingivalis*, periodontal disease, brown adipose tissue

## Abstract

Improvement of obesity is important for increasing longevity. The characteristics, size, and function of adipocytes are altered in patients with obesity. Adipose tissue is not only an energy storage but also an endocrine organ. Alteration of endocrine activities in adipose tissue, among them the functional decline of brown adipose tissue (BAT), is associated with obesity. Periodontal disease is a risk factor for systemic diseases since endotoxemia is caused by periodontal bacteria. However, the effect of periodontal disease on obesity remains unclear. Thus, this study aimed to investigate the effect of endotoxemia due to *Porphyromonas gingivalis*, a prominent cause of periodontal disease, on the BAT. Herein, endotoxemia was induced in 12-week-old C57BL/6J mice through intravenous injection of sonicated 10^8^ CFU of *P. gingivalis* (Pg) or saline (control [Co]) once. Eighteen hours later, despite no inflammatory M1 macrophage infiltration, inflammation-related genes were upregulated exclusively in the BAT of Pg mice compared with Co mice. Although no marked histological changes were observed in adipose tissues, expressions of genes related to lipolysis, *Lipe* and *Pnpla2* were downregulated after *P. gingivalis* injection in BAT. Furthermore, expression of *Pparg* and *Adipoq* was downregulated only in the BAT but not in the white adipose tissues, along with downregulation of *Ucp1* and *Cidea* expression, which are BAT-specific markers, in Pg mice. Microarray analysis of the BAT showed 106 differentially expressed genes between Co and Pg mice. Gene set enrichment analysis revealed that the cholesterol homeostasis gene set and PI3/Akt/mTOR signaling gene set in BAT were downregulated, whereas the TGF-β signaling gene set was enriched in Pg mice. Overall, intravenous injection of sonicated *P. gingivalis* altered the endocrine functions of the BAT in mice. This study indicates that endotoxemia by *P. gingivalis* potentially affects obesity by disrupting BAT function.

## Introduction

Obesity is a major public health concern worldwide and a risk factor for type 2 diabetes (Ozcan et al., 2004), and is also associated with multiple cancers (Key et al., 2003; MacInnis and English, 2006; Li et al., 2009; Lichtman, 2010). Furthermore, obesity itself increases mortality (Whitlock et al., 2009; Berrington de Gonzalez et al., 2010). Adipocytes in the adipose tissue produce various adipocytokines, including adiponectin and inflammatory cytokines (Hotamisligil, 2006; Kadowaki et al., 2006; Yoon et al., 2006), and play important roles in metabolism. Therefore, adipose tissue is considered an endocrine organ.

On the other hand, periodontal disease is also a global public health concern with its high prevalence (Pihlstrom et al., 2005). Periodontal disease results from chronic infections of periodontal bacteria, including *Porphyromonas gingivalis*, and leads to the destruction of bone and tissue around the teeth (Nassar et al., 2007). Periodontal disease results in not only tooth loss but also the aggravation of numerous types of systemic diseases including type 2 diabetes, cardiovascular disease, preterm low birth weight, and nonalcoholic fatty liver disease (Komazaki et al., 2017; Figuero et al., 2020; Genco and Borgnakke, 2020; Orlandi et al., 2020; Polak et al., 2020; Schenkein et al., 2020).

The association between obesity and periodontal disease has recently attracted increasing attention. Some studies reported that the prevalence of periodontitis is higher among individuals with obesity (Saito et al., 1998; Al-Zahrani et al., 2003; Katagiri et al., 2010). In addition, we previously reported that intravenous injections of sonicated *P. gingivalis* twice per week for 12 weeks to mice fed high-fat diet caused an increase in body weight and the accumulation of visceral and subcutaneous fat in mice (Sasaki et al., 2018). However, the direct effects of *P. gingivalis* on adipocytes remain unclear.

In this study, we investigated the effect of endotoxemia resulting from periodontal disease on adipose tissue upon intravenous injection of ultrasonicated *P. gingivalis* in mice, followed by a comprehensive analysis of gene expression in the brown adipose tissue (BAT).

## Materials and Methods

### Animals

C57BL/6J male mice (12-week-old; Sankyo Laboratory, Tokyo, Japan) were used in this study. The mice were provided ad libitum access to food and water throughout the study and housed under standard conditions on a 12-h light/dark (light: 8:00 to 20:00) cycle. Mice were randomly divided into two groups: those intravenously injected with 10^8^ CFU of sonicated *P. gingivalis* suspended in 100 μL of saline (Pg group) and those receiving only saline (control [Co] group). Visceral white adipose tissue (eWAT), subcutaneous white adipose tissue (iWAT), and BAT from interscapular fat were harvested 18 h after *P. gingivalis* injection. All protocols regarding animal use and euthanasia were reviewed and approved by the Animal Care Committee of the Experimental Animal Center at Tokyo Medical and Dental University (A2020–054A).

### *P. gingivalis* Culture

*P. gingivalis* (ATCC 33277) was cultured, as previously described (Sasaki et al., 2018; Udagawa et al., 2018), on trypticase soy agar (Difco Laboratories, Detroit, MI, USA) supplemented with 10% defibrinated horse blood, hemin, and menadione at 37°C under anaerobic conditions. After 48 h, *P. gingivalis* was inoculated in trypticase soy broth under anaerobic conditions and cultured at 37°C under anaerobic conditions to the mid-log phase, and then 10^9^ CFU/mL of the bacterial suspension was sonicated at an amplitude of 20 kHz for 5 min on ice using a Qsonica Q700 sonicator (Waken Btech, Kyoto, Japan).

### Quantitative Reverse-Transcription PCR Analysis

Total RNA was extracted from the eWAT, iWAT, and BAT (n = 7) using Trizol reagent (Invitrogen, Carlsbad, CA, USA) and NucleoSpin^®^ RNA kit (TaKaRa Bio, Shiga, Japan) in accordance with the manufacturer’s instructions. Five-hundred nanograms total RNA was reverse-transcribed to cDNA, using the PrimeScript™ RT Master Mix (TaKaRa Bio). Real-time PCR was performed using the Thermal Cycler Dice^®^ Real Time System II (TaKaRa Bio). PCR mixtures were prepared using TB Green Premix Ex Taq™ II (TaKaRa Bio). PCR was carried out in accordance with the manufacturer’s instructions. Gene expression levels were normalized to those of the reference gene, *Rn18s*. The PCR primers used herein are listed in **Supplementary Table S1**.

### Isolation of Stromal Vascular Fractions From the BAT

Stromal vascular fractions (SVFs) were isolated from the eWAT, iWAT, and BAT (n = 6) 18 h after *P. gingivalis* injection. Adipose tissues were finely minced and digested with collagenase (Wako, Osaka, Japan) with Krebs-Henseleit-HEPES buffer (pH 7.4) supplemented with 1% BSA and 0.2% glucose at 37°C for 60 min. Thereafter, the samples were strained through a 100-µm cell strainer and fractionated through centrifugation at 1,500 rpm for 5 min. The pellets were collected as cells in the SVF.

### Flow Cytometry Analysis

Erythrocytes were depleted using ACK lysing buffer (Lonza, Walkersville, MD, USA) for 5 min at room temperature. The cells were incubated with anti-mouse CD16/32 (2.4G2) (TONBO biosciences, San Diego, CA, USA) for 10 min and stained with anti-CD11b (M1/70), anti-CD11c (N418), and anti-CD206 (MR6F3) antibodies (Invitrogen) for 30 min at 4°C. The cells were analyzed using an Attune NxT flow cytometer (Thermo Fisher Scientific, Waltham, MA, USA). The data were analyzed using FlowJo software version 10.6.2 (BD Biosciences, San Jose, CA, USA). M1 or M2 macrophages were identified according to CD11b-positive/CD11c-positive/CD206-negative or CD11b-positive/CD11c-negative/CD206-positive cells, respectively (Fujisaka et al., 2009).

### Histological Analysis in Adipose Tissue

eWAT, iWAT, and BAT (n = 4) were collected 18 h after *P. gingivalis* injection and fixed in 4% paraformaldehyde in phosphate-buffered saline for 24 h. The tissues were then embedded in paraffin and then 5 µm sections were made. Specimens were stained with hematoxylin and eosin (HE) and examined under a light microscope (ECLIPSE Ni‐U, NIKON Corp., Tokyo, Japan) at ×200 magnification.

### Microarray Analysis

The Agilent Low Input Quick Amp Labeling kit (Agilent Technologies, Santa Clara, CA, USA) was used, in accordance with the manufacturer’s instructions, to generate complementary RNA (cRNA) from 200 ng total RNA for single-color (Cy3) microarray analysis (n = 4). Thereafter, cRNAs were analyzed through hybridization onto an Agilent SurePrint G3 Unrestricted Gene Expression 8 × 60 K Microarray (Agilent Technologies). Fluorescence signals were detected using the Agilent Microarray Scanner System (Agilent Technologies). Raw microarray data were extracted using Feature Extraction Software (ver. 11.0.1.1; Agilent Technologies).

### Statistical Analysis

Data distributions were analyzed using the Shapiro-Wilk test, revealing all datasets were normally distributed. Unpaired *t*-test was performed to compare the two groups. Data were analyzed using R (ver. 3.6.0). Microarray data were quantile-normalized, log_2_-transformed, and identified differentially expressed genes (DEGs) by using R with the Limma Bioconductor package (ver. 3.40.6) (Ritchie et al., 2015). Benjamin and Hochberg’s false discovery rate (FDR) was applied for multiple testing. DEGs were defined in accordance with an FDR q <0.1 and a |fold-change| >1.5. Overrepresentation enrichment analyses for DEGs were performed using the WEB-based Gene SeT AnaLysis Toolkit (http://www.webgestalt.org) (Wang et al., 2013) and the Database for Annotation, Visualization, and Integrated Discovery (DAVID) (http://david.abcc.ncifcrf.gov/) using the Gene Ontology (GO) and KEGG pathway databases. Gene set enrichment analysis (GSEA) (http://software.broadinstitute.org/gsea/index.jsp) (Subramanian et al., 2005) was performed using hallmark gene sets (Liberzon et al., 2015).

## Results

### *P. gingivalis* Injection Increased Inflammation-Related mRNA Expression in the BAT

**Figure 1:**
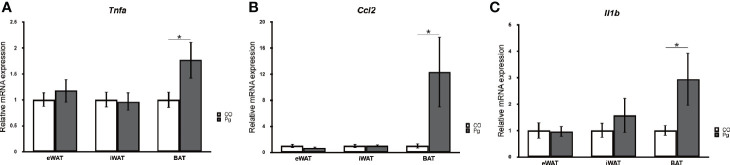
Quantitative reverse-transcription PCR analysis in the adipose tissues at 18 h after *P. gingivalis* injection (n = 7). **(A)** *Tnfa*, **(B)** *Ccl2*, **(C)** *Il1b* expression in the adipose tissues (means ± SE). *P < 0.05.

The mRNA expression levels of tumor necrosis factor-α (*Tnfa*), monocyte chemotactic protein-1 (MCP-1) (*Ccl2*), and interleukin 1 beta (*Il1b*) were not significantly altered in the eWAT and iWAT following *P. gingivalis* injection. However, the expression of *Tnfa* and *Il1b* was significantly upregulated in the BAT in Pg mice compared to the Co mice (**Figures 1A, B**). Interestingly, the mRNA expression level of *Ccl2* was dramatically increased only in the BAT following *P. gingivalis* injection (**Figure 1C**).

### No Alteration in Macrophage Infiltration Was Observed in SVF From Adipose Tissues

**Figure 2:**
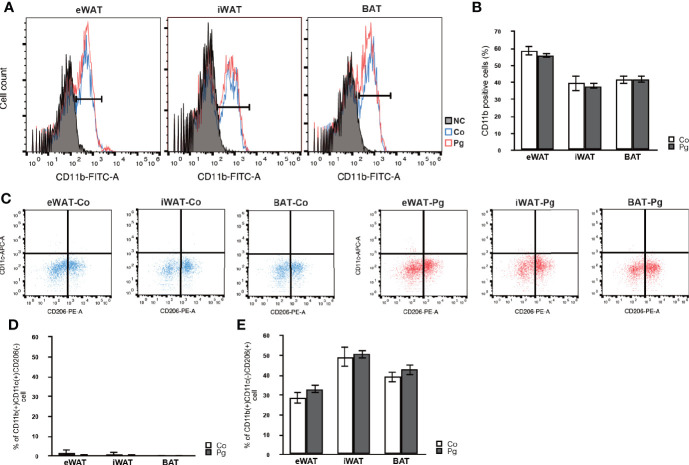
Flow cytometry analysis in cells from the SVF of adipose tissues at 18 h after *P. gingivalis* injection (n = 6). **(A)** Representative flow cytometry results. Black; negative control. Blue; Co mice. Red; Pg mice. **(B)** The percentage of CD11b-positive cells in the cells at SVF from adipose tissues. **(C)** Representative results of flow cytometry are shown. CD11b-positive/CD11c-positive/CD206-negative cells are defined as M1 macrophages. CD11b-positive/CD11c-negative/CD206-positive cells are defined as M2 macrophages. **(D)** The percentage of M1 macrophage in the CD11b-positive cells at SVF from adipose tissues. **(E)** The percentage of M2 macrophage in the CD11b-positive cells at SVF from adipose tissues.

Macrophage infiltration in the SVF from the eWAT, iWAT, and BAT was evaluated through flow cytometry, respectively. *P. gingivalis* injection caused no significant changes in the percentage of CD11b positive cells in the SVF from eWAT, iWAT, and BAT (**Figures 2A, B**). Few M1 macrophages were observed in eWAT, iWAT, and BAT of Co and Pg mice (**Figure 2C**). The percentage of M2 macrophages in CD11b+ cells was 28.7% and 33.1% in the SVF from eWAT of Co and Pg mice, 49.3% and 50.7% in the SVF from iWAT of Co and Pg mice, and 39.2% and 42.9% in the SVF from BAT of Co and Pg mice, respectively. The percentage of M1 and M2 macrophages in eWAT, iWAT, and BAT did not significantly differ between Co and Pg mice (**Figures 2D, E**).

### *P. gingivalis* Injection Downregulated mRNA Expression of Genes Related to Lipolysis and Metabolism in BAT

**Figure 3:**
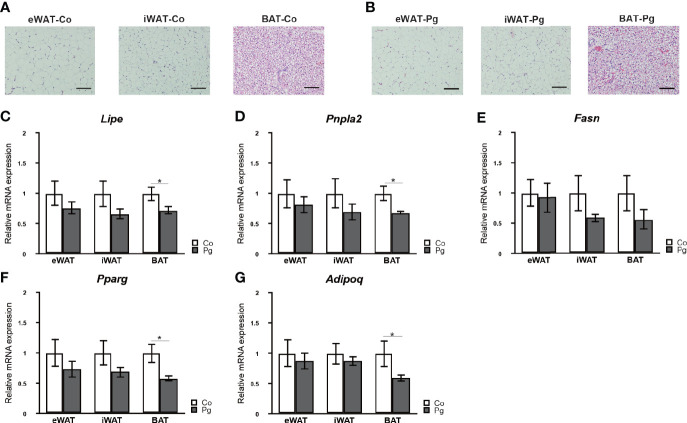
Evaluation of adipose tissues at 18 h after *P. gingivalis* injection. Representative HE staining of adipose tissues from **(A)** Co, **(B)** Pg mice (row magnification × 200, black bar = 100 μm) (n = 4). Quantitative reverse-transcription PCR analysis in the adipose tissues at 18 h after *P. gingivalis* injection (n = 7). **(C)** *Lipe*, **(D)** *Pnpla2*, **(E)** *Fasn*, **(F)** *Pparg*, **(G)** *Adipoq* expression in the adipose tissues (means ± SE). *P < 0.05.

Although no marked histological changes were observed after *P. gingivalis* injection in adipose tissues (**Figures 3A, B**), the expression of lipase, hormone sensitive (*Lipe*) and patatin-like phospholipase domain containing 2 (*Pnpla2*) in the BAT of Pg mice were significantly downregulated compared to those of Co mice. Furthermore, the expression of fatty acid synthase (*Fasn*) in the BAT tended to be decreased in Pg mice. There was no significant difference in the expression of *Lipe*, *Pnpla2*, and *Fasn* in eWAT and iWAT between Co and Pg mice (**Figures 3C–E**).

Peroxisome proliferator activated receptor gamma (*Pparg*) and adiponectin (*Adipoq*) expression levels were not significantly altered in the eWAT and iWAT following *P. gingivalis* injection. However, the mRNA expression of *Pparg* in the BAT of Pg mice was significantly decreased compared to Co mice (**Figure 3F**). Moreover, *P. gingivalis* injection significantly downregulated *Adipoq* expression in BAT (**Figure 3G**).

### *P. gingivalis* Injection Altered Gene Expression Patterns in the BAT

To investigate changes in gene expression in the BAT at 18 h after injection of sonicated *P. gingivalis*, microarray analysis was performed to obtain a comprehensive overview of gene expression profiles. All microarray data herein are available in the Gene Expression Omnibus database (www.ncbi.nlm.nih.gov/geo) under GSE 153516.

**Figure 4:**
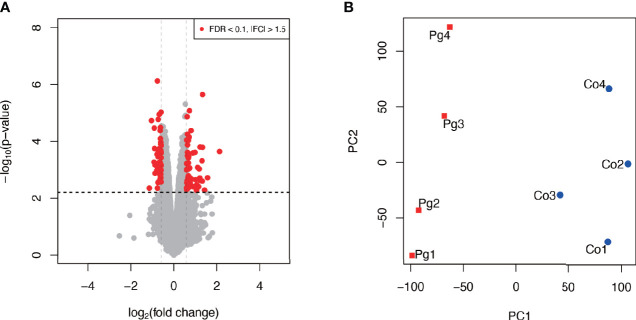
Microarray analysis in the brown adipose tissue between Co and Pg mice at 18 h after *P. gingivalis* injection (n = 4). **(A)** Volcano plot; red plots show genes with FDR q < 0.1 and |fold change| > 1.5. **(B)** Principal component analysis.

As shown in **Figure 4A**, among 106 DEGs (|fold change| > 1.5 and q < 0.1), 60 genes were upregulated, and 46 genes were downregulated. Gene expression patterns substantially differed between Co and Pg mice (**Figure 4B**).

### *P. gingivalis* Injection Altered BAT Metabolism

**Figure 5:**
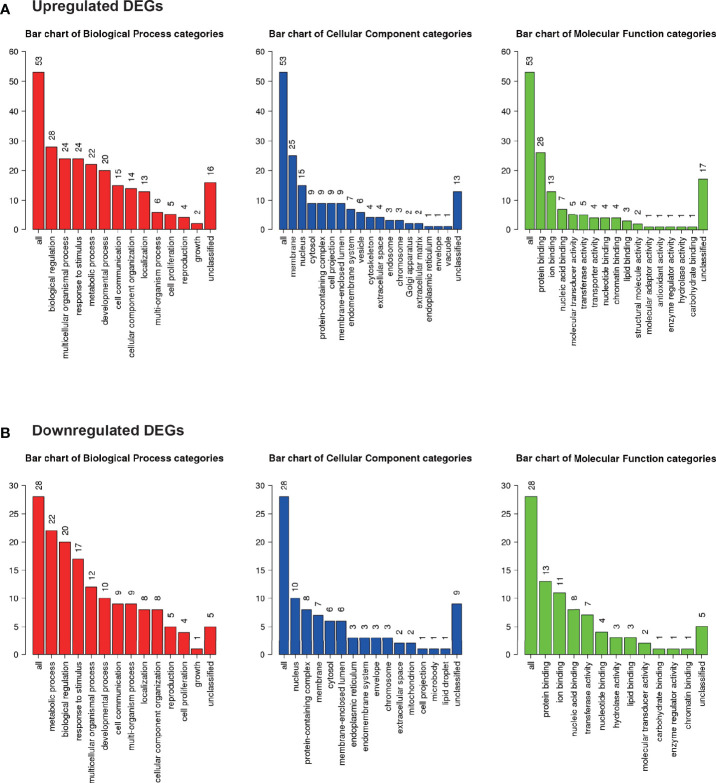
Microarray analysis in the brown adipose tissue between Co and Pg mice (n = 4). Gene Ontology analysis in **(A)** upregulated DEGs and **(B)** downregulated DEGs.

Gene ontology was assessed using GO slim for upregulated (**Figure 5A**) and downregulated (**Figure 5B**) DEGs. Notably, 42% of upregulated DEGs with GO terms were classified under “metabolic process” in the biological process category, whereas 79% of downregulated DEGs were classified under “metabolic process”.

**Figure 6:**
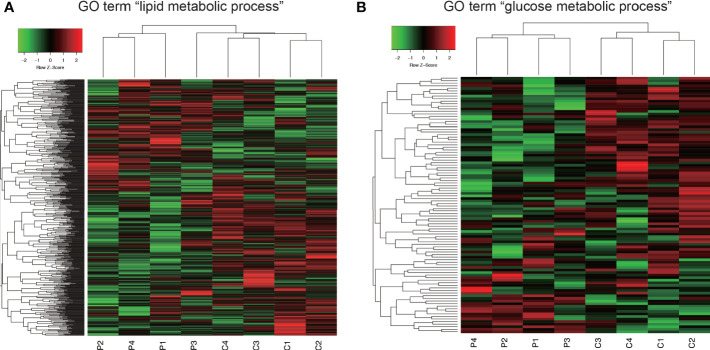
Comprehensive evaluation of gene expression in brown adipose tissue between Co and Pg mice at 18 h after *P. gingivalis* injection. **(A)** Heatmap of genes extracted according to the GO term including “lipid metabolic process”, **(B)** Heatmap of genes extracted according to the GO term including “glucose metabolic process”.

In GO analysis, genes were extracted in accordance with the GO term “lipid metabolic process” or “glucose metabolic process” from all detectable genes through microarray analysis. In total, 615 genes were identified under the GO term “lipid metabolic process” (**Figure 6A**), and 100 genes contained GO term “glucose metabolic process” (**Figure 6B**). As shown in **Figure 6**, Co and Pg mice presented markedly different gene expression patterns for lipid metabolic process and glucose metabolic process.

**Figure 7:**
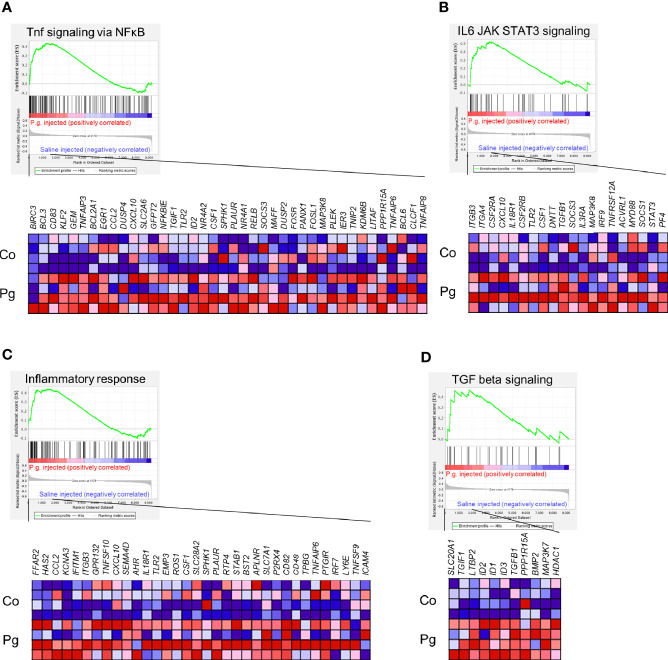
Gene sets enriched in Pg mice at 18 h after *P. gingivalis* injection (n = 4). Gene sets about **(A)** TNFα signaling *via* NFκB, **(B)** IL6/JAK/STAT3 signaling, **(C)** Inflammatory response, and **(D)** TGF-β signaling. A heatmap provided illustrating gene expression levels for each gene in the core enrichment subset (blue: low, red: high). NES, normalized enrichment score.

**Figure 8:**
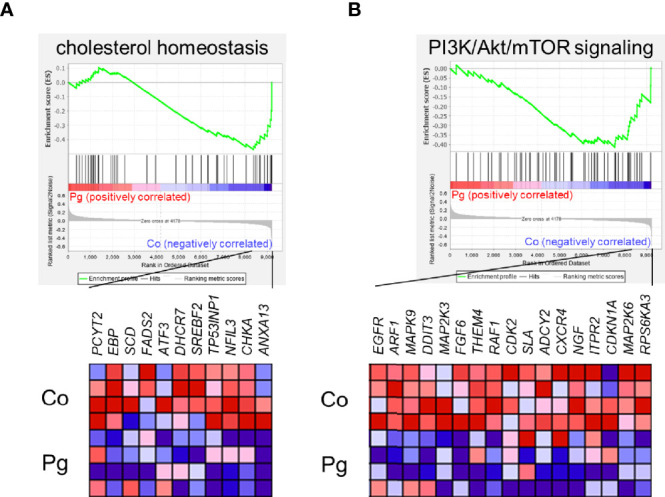
Gene sets downregulated in Pg mice compared to Co mice at 18 h after *P. gingivalis* injection (n = 4). Gene sets about **(A)** cholesterol homeostasis, and **(B)** PI3K/Akt/mTOR signaling. A heatmap provided illustrating gene expression levels for each gene in the core enrichment subset (blue: low, red: high). NES, normalized enrichment score.

GSEA was performed using hallmark gene sets to evaluate the differences in mRNA expression patterns in the BAT between Co and Pg mice. Upregulated gene sets with an FDR q < 0.15 are listed in **Table 1**. Several inflammation-related gene sets were enriched in the BAT of Pg mice, including the TNFα signaling *via* NFκB gene set (**Figure 7A**; normalized enrichment score (NES) = 1.73, q = 0.013), IL6/JAK/STAT3 signaling gene set (**Figure 7B**; NES = 1.71, q = 0.013) (**Figure 7B**), and inflammatory response gene set (**Figure 7C**; NES = 1.69, q = 0.014). Furthermore, TGF beta; signaling gene set was also enriched (**Figure 7D**; NES = 1.43, q = 0.066). However, only 3 downregulated gene sets with an FDR q < 0.15 were identified (**Table 2**). The cholesterol homeostasis gene set (**Figure 8A**; NES = −1.56, q = 0.110) and PI3K/Akt/mTOR signaling gene set (**Figure 8B**; NES = −1.49, q = 0.149) in BAT was downregulated in Pg mice.

**Table 1:** Gene sets enriched in Pg mice at 18 h after *P. gingivalis* injection.

Gene set	Size	NES	normal p-value	FDR q-value
allograft rejection	94	2.29	<0.001	<0.001
interferon alpha response	65	2.14	<0.001	<0.001
interferon gamma response	129	1.98	<0.001	0.001
TNFα signaling *via* NFκB	121	1.73	<0.001	0.013
IL6/JAK/STAT3 signaling	44	1.71	0.004	0.013
inflammatory response	96	1.69	0.002	0.014
coagulation	75	1.68	0.002	0.013
IL2-STAT5 signaling	114	1.68	0.002	0.011
apical junction	112	1.55	0.002	0.035
epithelial mesenchymal transition	117	1.49	0.013	0.051
KRAS signaling up	120	1.45	0.014	0.063
TGF beta signaling	31	1.43	0.042	0.066
estrogen response late	108	1.38	0.045	0.093
complement	115	1.37	0.039	0.093
angiogenesis	16	1.35	0.150	0.098

**Table 2:** Gene sets downregulated in Pg mice at 18 h after *P. gingivalis* injection.

Gene set	Size	NES	normal p-value	FDR q-value
KRAS signaling dn	74	−1.66	0.004	0.086
cholesterol homeostasis	46	−1.56	0.010	0.110
PI3K/Akt/mTOR signaling	61	−1.49	0.020	0.149

### *P. gingivalis* Injection Downregulated the Transcript Levels of Several Relevant BAT Marker Genes and Endocrine-Derived Factors from BAT

**Figure 9:**
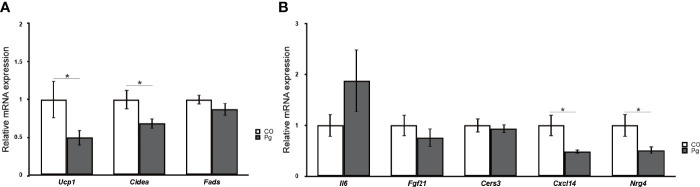
Quantitative reverse-transcription PCR analysis in the brown adipose tissues at 18 h after *P. gingivalis* injection (n = 7). **(A)** *Ucp1*, *Cidea*, *Fads*, **(B)** *Il6*, *Fgf21*, *Cers3*, *Cxcl14*, *Nrg4* expression in the brown adipose tissues (means ± SE). *P < 0.05.

The expression levels of uncoupling protein 1 (*Ucp1*) and cell death-inducing DNA fragmentation factor, alpha subunit-like effector A (*Cidea*), which are BAT markers, were significantly downregulated in the BAT of Pg mice compared to those in Co mice, although the expression levels of fatty acid desaturase 3 (*Fads3*), a white adipocyte tissue marker, was comparable between Co and Pg mice (**Figure 9A**). Interleukin 6 (*Il6*) expression in the BAT of Pg mice tended to be increased in Pg mice. The expression of fibroblast growth factor 21 (*Fgf21*) and ceramide synthase 3 (T3) (*Cers3*) was not significantly altered in the BAT after *P. gingivalis* injection. However, chemokine (C-X-C motif) ligand 14 (*Cxcl14*) and neuregulin 4 (*Nrg4*) expression were significantly downregulated in the BAT of Pg mice compared to those of Co mice (**Figure 9B**).

## Discussion

A previous epidemiological study showed an association between periodontal disease and obesity in 1998 (Saito et al., 1998). In addition, another paper showed the causal effects of *P. gingivalis* infection that increased body weight in mice (Yoneda et al., 2012). We also reported that multiple intravenous injections of sonicated *P. gingivalis* increased body weight and the accumulation of visceral and subcutaneous fats in mice fed high-fat diet (Sasaki et al., 2018). However, the mechanisms, especially the direct effects of *P. gingivalis* infection on adipocytes still remained unclear, and this study was conducted to address this issue.

In this study, endotoxemia was induced through an intravenous injection of ultrasonicated *P. gingivalis* to simulate endotoxemia by periodontal disease. Endotoxemia occurs in patients with severe periodontal diseases (Ide et al., 2004; Forner et al., 2006; Tonetti et al., 2007). *P. gingivalis* is a representative periodontal pathogen and contains various virulence factors including lipopolysaccharide, fimbriae, and enzymes (Kolenbrander et al., 2002). In addition, we have recently reported that only *P. gingivalis* infection, but not other periodontopathic bacteria including *Aggregatibacter actinomycetemcomitans* and *Fusobacterium nucleatum*, is significantly associated with intramuscular adipose tissue content in the lumbar muscles (Watanabe et al., 2020). Based on the above results, we focused on *P. gingivalis* infection. Although only a single oral administration of periodontal pathogen could not cause periodontitis in mice (Lalla et al., 1998; Lalla et al., 2000), the purpose of this study was to observe the differences in gene expression due to endotoxemia induced by *P. gingivalis* in adipocytes. Thus, we used mice fed normal chow and performed only a single injection of *P. gingivalis*.

Interscapular fat is different from other adipose tissues, such as visceral and subcutaneous adipose tissues. Interscapular fat is rich in brown adipocytes; whereas, visceral and subcutaneous adipose tissues are rich in white adipocytes (Wang and Seale, 2016). BAT differs from white adipose tissue which works as an energy storage, and BAT can generate heat and expend energy by consuming glucose and fatty acid. (Nedergaard and Cannon, 2010). Since several reports have shown that BAT is present in adult humans (Cypess et al., 2009; Saito et al., 2009), numerous studies have been performed to prevent and improve obesity and metabolic diseases by focusing on the BAT (van der Lans et al., 2013; Yoneshiro et al., 2013; Hiraike et al., 2017).

Macrophages themselves produce inflammatory cytokines including TNF-α and CCL2. M1 macrophages produce inflammatory cytokines whereas M2 macrophages suppress inflammation (Lumeng et al., 2007). Interestingly, *Tnfa* and *Ccl2* expression were upregulated exclusively in the BAT after *P. gingivalis* injection without infiltration of inflammatory M1 macrophages, which suggests that *P. gingivalis* injection altered adipocytokine production in non-macrophage cells from the BAT, potentially brown adipocytes. The downregulation of *Lipe* and *Pnpla2* expression in the BAT suggested that *P. gingivalis* injection altered lipid metabolism in BAT. However, only a single *P. gingivalis* injection did not alter adipocyte size, although there is a possibility that multiple and long-term *P. gingivalis* injections can cause hypertrophy of adipocytes in BAT. *Adipoq* and *Pparg* expression in BAT were downregulated by *P. gingivalis* injection, even though there was no significant difference in the expression of *Adipoq* and *Pparg* in eWAT and iWAT between Co and Pg mice. Adiponectin is an adipocyte-derived hormone that improves dyslipidemia and insulin resistance (Yamauchi et al., 2001), and activation of the adiponectin receptor can improve obesity-related diseases (Okada-Iwabu et al., 2013). *Pparg* regulates glucose and lipid metabolism (Berger and Moller, 2002) and directly regulates numerous genes involved in the functions of adipocytes, lipid transport, insulin signaling, and adipokine production (Lehrke and Lazar, 2005). Agonist PPARγ ligands are already used for the treatment of type 2 diabetes (Lehmann et al., 1995). Furthermore, adiponectin expression is controlled by PPARγ. In obesity, inflammation is known to inhibit PPARγ expression and function, thus inhibiting its direct targets such as adiponectin (Guilherme et al., 2008). Therefore, our result about downregulation of *Adipoq* could be an indirect effect of downregulation in *Pparg* expression.

Microarray analysis also revealed the possibility of alterations in glucose and lipid metabolisms. GSEA supports these results since the “cholesterol homeostasis” and “PI3K/Akt/mTOR” gene sets were downregulated. Downregulation of PI3K/Akt/mTOR signaling gene set indicates downregulation of insulin signaling (Taniguchi et al., 2006). As previously reported, BAT is also associated with glucose homeostasis and insulin sensitivity. Therefore, this result suggests that *P. gingivalis* injection may cause a decrease in glucose homeostasis and insulin sensitivity in brown adipocytes (Stanford et al., 2013). Cholesterol imbalance has been recognized as a feature of obese adipocytes (Yu et al., 2010). Downregulation of cholesterol homeostasis gene set suggested that this can be extrapolated to obese mice. As previously reported, TGF-β levels correlate with obesity in mice (Samad et al., 1997; Samad et al., 1999) and humans (Alessi et al., 2000; Fain et al., 2005; Lin et al., 2009). Moreover, systemic blockade of TGF-β signaling protected mice from obesity, diabetes, and hepatic steatosis in a previous study (Yadav et al., 2011). Thus, upregulation of TGF-β signaling also indicates that the BAT in Pg mice may be the similar state to that in obese mice. In addition, we showed differential gene expression patterns for lipid metabolic process and glucose metabolic process between Co and Pg mice, and the percentage of downregulated “metabolic process” DEGs in the biological process category were larger than upregulated DEGs. These results suggested that metabolic function in the BAT was altered by *P. gingivalis* injection. On the other hand, inflammation-related gene sets, TNF-α signaling *via* NF-κB gene set, IL6/JAK/STAT3 signaling gene set, and inflammatory response gene set, were enriched in the BAT, concurrent with the results of quantitative PCR, and supported evidence regarding inflammation in the BAT after *P. gingivalis* injection.

Surprisingly, just a single intravenous injection of *P. gingivalis* downregulated the expression of *Ucp1* and *Cidea* in the BAT. UCP1 is specific and responsible for heat production in the BAT and is involved in ATP synthesis (Nedergaard et al., 2005). Loss of UCP1 function enhanced obesity in mice on a high-fat diet (Feldmann et al., 2009). In addition, *Cidea* is a specific and important gene in BAT that regulates adipocyte differentiation and tight coupling of lipolysis and lipogenesis (Wang and Seale, 2016). CIDEA, a lipid droplet protein, and UCP1 are regulated by PPARγ (Berger and Moller, 2002; Puri et al., 2008). Based on these results and the fact that BAT consumes high amount of glucose (Shibata et al., 1989; Liu et al., 1994), we suggest that the intravenous injection of sonicated *P. gingivalis* downregulated the expression of *Pparg* followed by *Ucp1* and *Cidea*, potentially altering glucose and lipid metabolism. In this study, *Il6* expression tended to be increased in BAT from Pg mice. IL-6 is widely recognized as a batokine, it is a BAT-derived secretable factor with pleiotropic actions, mediating glucose homeostasis, insulin sensitivity, and thermogenesis in BAT (Stanford et al., 2013; Villarroya et al., 2017), which is against classic inflammation-mediated effects and performs relevant metabolic functions by increasing insulin sensitivity in the muscle, promoting browning of WAT and M2 macrophage polarization (Mauer et al., 2014) or more recently, mediating stress responses and liver gluconeogenesis (Qing et al., 2020). In addition, just a single intravenous injection of *P. gingivalis* downregulated the expression of *Cxcl14* and *Nrg4* in the BAT. These endocrine-derived factors from BAT can affect distant organs. CXCL14 from BAT appears to influence the recruitment of M2 macrophages to subcutaneous WAT. In addition, a lack of CXCL14 in BAT correlated with an impairment of BAT function (Cereijo et al., 2018). Nrg4 from BAT acts on the liver to attenuate hepatic lipogenic signaling (Wang et al., 2014). Our results support the previous report that *P. gingivalis* infection promoted liver steatosis in mice (Sasaki et al., 2018). These results suggested that endotoxemia due to *P. gingivalis* directly affects the endocrine function in the BAT.

We previously reported that intravenous injections of sonicated *P. gingivalis*; twice per week for 12 weeks, increased visceral and subcutaneous fat, impaired glucose tolerance and insulin resistance, and resulted in liver steatosis and inflammation in mice administered a high-fat diet (Sasaki et al., 2018). In addition, a few other studies have reported the effect of *P. gingivalis* on adipocytes. Adiponectin was significantly downregulated in 3T3-L1 adipocytes treated with *P. gingivalis* LPS rather than with *Escherichia coli* LPS (Le Sage et al., 2017). Furthermore, successful periodontal treatment increased serum adiponectin levels in patients with type 2 diabetes (Bharti et al., 2013). Although the main contributor of systemic adiponectin level is the WATs, not the BAT (Giralt et al., 2016), in our results and from previous reports may indicate circulation levels of adiponectin may be different between Co and Pg mice. However, we feel it is difficult to detect differences in only one time point experiment at 18 h after *P. gingivalis* injection. Thus, further studies, especially long term and with multiple *P. gingivalis* injections evaluating systemic effects by altering BAT metabolism are required to determine the effect of periodontal infection on adipose tissues. In addition, BAT function is mainly controlled by brown adipose tissues, but also is controlled by several other cells including preadipocyes, endothelial cells, immune cells, and sympathetic neurons (Cannon and Nedergaard, 2010; Villarroya et al., 2018). Therefore, it is unclear whether our results are direct effects of *P. gingivalis* endotoxemia on brown adipocytes. Further *in vitro* studies are required to determine the specific pathways of *P. gingivalis* endotoxemia on isolated brown adipocytes.

In conclusion, endotoxemia due to sonicated *P. gingivalis* may directly affect the endocrine function in the BAT. Moreover, periodontal bacterial infections potentially cause alterations in BAT functional markers and immunometabolic characteristics. To our knowledge, this is the first study to comprehensively evaluate gene expression profiles in BAT after endotoxemia-induction through *P. gingivalis* injection in mice. The present results suggest that endotoxemia by *P. gingivalis* affects obesity by disrupting adipocyte function.

## Data Availability Statement

The datasets presented in this study can be found in online repositories. The names of the repository/repositories and accession number(s) can be found below: https://www.ncbi.nlm.nih.gov/geo/, GSE 153516.

## Ethics Statement

The animal study was reviewed and approved by the Animal Care Committee of the Experimental Animal Center at Tokyo Medical and Dental University.

## Author Contributions

MH and YO performed most of the experiments and wrote the first draft of the manuscript. HNii, KM, YT, SY, TShim, NS, SM, TShib, TH, HTo, HTa, and TI assisted in some studies and reviewed the manuscript. SK and HNit supervised all the studies and the writing of the manuscript. All authors contributed to the article and approved the submitted version.

## Funding

This work was supported by the Japan Society for the Promotion of Science (20H03863 to SK, 19K24062 to NS, 19K18989 to SM, and 19K19016 to TShib).

## Conflict of Interest

The authors declare that the research was conducted in the absence of any commercial or financial relationships that could be construed as a potential conflict of interest.

## References

[B1] Alessi M. C. Bastelica D. Morange P. Berthet B. Leduc I. Verdier M. (2000). Plasminogen activator inhibitor 1, transforming growth factor-beta1, and BMI are closely associated in human adipose tissue during morbid obesity. Diabetes 49 (8), 1374–1380. 10.2337/diabetes.49.8.1374 10923640

[B2] Al-Zahrani M. S. Bissada N. F. Borawskit E. A. (2003). Obesity and periodontal disease in young, middle-aged, and older adults. J. Periodontol. 74 (5), 610–615. 10.1902/jop.2003.74.5.610 12816292

[B3] Berger J. Moller D. E. (2002). The Mechanisms of Action of PPARs. Annu. Rev. Med. 53 (1), 409–435. 10.1146/annurev.med.53.082901.104018 11818483

[B4] Berrington de Gonzalez A. Hartge P. Cerhan J. R. Flint A. J. Hannan L. MacInnis R. J. (2010). Body-mass index and mortality among 1.46 million white adults. N Engl. J. Med. 363 (23), 2211–2219. 10.1056/NEJMoa1000367 21121834 PMC3066051

[B5] Bharti P. Katagiri S. Nitta H. Nagasawa T. Kobayashi H. Takeuchi Y. (2013). Periodontal treatment with topical antibiotics improves glycemic control in association with elevated serum adiponectin in patients with type 2 diabetes mellitus. Obes. Res. Clin. Pract. 7 (2), e129–e138. 10.1016/j.orcp.2011.11.005 24331774

[B6] Cannon B. Nedergaard J. (2010). Metabolic consequences of the presence or absence of the thermogenic capacity of brown adipose tissue in mice (and probably in humans). Int. J. Obes. (Lond) 34 Suppl 1, S7–16. 10.1038/ijo.2010.177 20935668

[B7] Cereijo R. Gavaldà-Navarro A. Cairó M. Quesada-López T. Villarroya J. Morón-Ros S. (2018). CXCL14, a Brown Adipokine that Mediates Brown-Fat-to-Macrophage Communication in Thermogenic Adaptation. Cell Metab. 28 (5), 750–763.e756. 10.1016/j.cmet.2018.07.015 30122557

[B8] Cypess A. M. Lehman S. Williams G. Tal I. Rodman D. Goldfine A. B. (2009). Identification and importance of brown adipose tissue in adult humans. N Engl. J. Med. 360 (15), 1509–1517. 10.1056/NEJMoa0810780 19357406 PMC2859951

[B9] Fain J. N. Tichansky D. S. Madan A. K. (2005). Transforming growth factor beta1 release by human adipose tissue is enhanced in obesity. Metabolism 54 (11), 1546–1551. 10.1016/j.metabol.2005.05.024 16253647

[B10] Feldmann H. M. Golozoubova V. Cannon B. Nedergaard J. (2009). UCP1 Ablation Induces Obesity and Abolishes Diet-Induced Thermogenesis in Mice Exempt from Thermal Stress by Living at Thermoneutrality. Cell Metab. 9 (2), 203–209. 10.1016/j.cmet.2008.12.014 19187776

[B11] Figuero E. Han Y. W. Furuichi Y. (2020). Periodontal diseases and adverse pregnancy outcomes: Mechanisms. Periodontol. 2000 83 (1), 175–188. 10.1111/prd.12295 32385886

[B12] Forner L. Nielsen C. H. Bendtzen K. Larsen T. Holmstrup P. (2006). Increased plasma levels of IL-6 in bacteremic periodontis patients after scaling. J. Clin. Periodontol. 33 (10), 724–729. 10.1111/j.1600-051x.2006.00964.x 16901299

[B13] Fujisaka S. Usui I. Bukhari A. Ikutani M. Oya T. Kanatani Y. (2009). Regulatory mechanisms for adipose tissue M1 and M2 macrophages in diet-induced obese mice. Diabetes 58 (11), 2574–2582. 10.2337/db08-1475 19690061 PMC2768159

[B14] Genco R. J. Borgnakke W. S. (2020). Diabetes as a potential risk for periodontitis: association studies. Periodontol. 2000 83 (1), 40–45. 10.1111/prd.12270 32385881

[B15] Giralt M. Cereijo R. Villarroya F. (2016). Adipokines and the Endocrine Role of Adipose Tissues. Handb. Exp. Pharmacol. 233, 265–282. 10.1007/164_2015_6 25903415

[B16] Guilherme A. Virbasius J. V. Puri V. Czech M. P. (2008). Adipocyte dysfunctions linking obesity to insulin resistance and type 2 diabetes. Nat. Rev. Mol. Cell Biol. 9 (5), 367–377. 10.1038/nrm2391 18401346 PMC2886982

[B17] Hiraike Y. Waki H. Yu J. Nakamura M. Miyake K. Nagano G. (2017). NFIA co-localizes with PPARγ and transcriptionally controls the brown fat gene program. Nat. Cell Biol. 19 (9), 1081–1092. 10.1038/ncb3590 28812581 PMC5885759

[B18] Hotamisligil G. S. (2006). Inflammation and metabolic disorders. Nature 444 (7121), 860–867. 10.1038/nature05485 17167474

[B19] Ide M. Jagdev D. Coward P. Y. Crook M. Barclay G. R. Wilson R. F. (2004). The Short-Term Effects of Treatment of Chronic Periodontitis on Circulating Levels of Endotoxin, C-Reactive Protein, Tumor Necrosis Factor-α, and Interleukin-6. J. Periodontol. 75 (3), 420–428. 10.1902/jop.2004.75.3.420 15088881

[B20] Kadowaki T. Yamauchi T. Kubota N. Hara K. Ueki K. Tobe K. (2006). Adiponectin and adiponectin receptors in insulin resistance, diabetes, and the metabolic syndrome. J. Clin. Invest. 116 (7), 1784–1792. 10.1172/jci29126 16823476 PMC1483172

[B21] Katagiri S. Nitta H. Nagasawa T. Izumi Y. Kanazawa M. Matsuo A. (2010). High prevalence of periodontitis in non-elderly obese Japanese adults. Obes. Res. Clin. Pract. 4 (4), e247–e342. 10.1016/j.orcp.2010.08.005 24345696

[B22] Key T. J. Appleby P. N. Reeves G. K. Roddam A. Dorgan J. F. Longcope C. (2003). Body mass index, serum sex hormones, and breast cancer risk in postmenopausal women. J. Natl. Cancer Inst. 95 (16), 1218–1226. 10.1093/jnci/djg022 12928347

[B23] Kolenbrander P. E. Andersen R. N. Blehert D. S. Egland P. G. Foster J. S. Palmer R. J. (2002). Communication among Oral Bacteria. Microbiol. Mol. Biol. Rev. 66 (3), 486–505. 10.1128/mmbr.66.3.486-505.2002 12209001 PMC120797

[B24] Komazaki R. Katagiri S. Takahashi H. Maekawa S. Shiba T. Takeuchi Y. (2017). Periodontal pathogenic bacteria, Aggregatibacter actinomycetemcomitans affect non-alcoholic fatty liver disease by altering gut microbiota and glucose metabolism. Sci. Rep. 7 (1), 13950. 10.1038/s41598-017-14260-9 29066788 PMC5655179

[B25] Lalla E. Lamster I. B. Feit M. Huang L. Schmidt A. M. (1998). A murine model of accelerated periodontal disease in diabetes. J. Periodontal. Res. 33 (7), 387–399. 10.1111/j.1600-0765.1998.tb02335.x 9842504

[B26] Lalla E. Lamster I. B. Feit M. Huang L. Spessot A. Qu W. (2000). Blockade of RAGE suppresses periodontitis-associated bone loss in diabetic mice. J. Clin. Invest. 105 (8), 1117–1124. 10.1172/jci8942 10772656 PMC300834

[B27] Le Sage F. Meilhac O. Gonthier M.-P. (2017). Porphyromonas gingivalis lipopolysaccharide induces pro-inflammatory adipokine secretion and oxidative stress by regulating Toll-like receptor-mediated signaling pathways and redox enzymes in adipocytes. Mol. Cell. Endocrinol. 446, 102–110. 10.1016/j.mce.2017.02.022 28216438

[B28] Lehmann J. M. Moore L. B. Smith-Oliver T. A. Wilkison W. O. Willson T. M. Kliewer S. A. (1995). An antidiabetic thiazolidinedione is a high affinity ligand for peroxisome proliferator-activated receptor gamma (PPAR gamma). J. Biol. Chem. 270 (22), 12953–12956. 10.1074/jbc.270.22.12953 7768881

[B29] Lehrke M. Lazar M. A. (2005). The Many Faces of PPARγ. Cell 123 (6), 993–999. 10.1016/j.cell.2005.11.026 16360030

[B30] Li D. Morris J. S. Liu J. Hassan M. M. Day R. S. Bondy M. L. (2009). Body mass index and risk, age of onset, and survival in patients with pancreatic cancer. Jama 301 (24), 2553–2562. 10.1001/jama.2009.886 19549972 PMC2760963

[B31] Liberzon A. Birger C. Thorvaldsdottir H. Ghandi M. Mesirov J. P. Tamayo P. (2015). The Molecular Signatures Database (MSigDB) hallmark gene set collection. Cell Syst. 1 (6), 417–425. 10.1016/j.cels.2015.12.004 26771021 PMC4707969

[B32] Lichtman M. A. (2010). Obesity and the risk for a hematological malignancy: leukemia, lymphoma, or myeloma. Oncologist 15 (10), 1083–1101. 10.1634/theoncologist.2010-0206 20930095 PMC3227901

[B33] Lin Y. Nakachi K. Ito Y. Kikuchi S. Tamakoshi A. Yagyu K. (2009). Variations in serum transforming growth factor-beta1 levels with gender, age and lifestyle factors of healthy Japanese adults. Dis. Markers 27 (1), 23–28. 10.3233/dma-2009-0643 19822955 PMC3834674

[B34] Liu X. Pérusse F. Bukowiecki L. J. (1994). Chronic norepinephrine infusion stimulates glucose uptake in white and brown adipose tissues. Am. J. Physiol. 266 (3 Pt 2), R914–R920. 10.1152/ajpregu.1994.266.3.R914 8160886

[B35] Lumeng C. N. Bodzin J. L. Saltiel A. R. (2007). Obesity induces a phenotypic switch in adipose tissue macrophage polarization. J. Clin. Invest. 117 (1), 175–184. 10.1172/jci29881 17200717 PMC1716210

[B36] MacInnis R. J. English D. R. (2006). Body size and composition and prostate cancer risk: systematic review and meta-regression analysis. Cancer Causes Control 17 (8), 989–1003. 10.1007/s10552-006-0049-z 16933050

[B37] Mauer J. Chaurasia B. Goldau J. Vogt M. C. Ruud J. Nguyen K. D. (2014). Signaling by IL-6 promotes alternative activation of macrophages to limit endotoxemia and obesity-associated resistance to insulin. Nat. Immunol. 15 (5), 423–430. 10.1038/ni.2865 24681566 PMC4161471

[B38] Nassar H. Kantarci A. van Dyke T. E. (2007). Diabetic periodontitis: a model for activated innate immunity and impaired resolution of inflammation. Periodontol. 2000 43, 233–244. 10.1111/j.1600-0757.2006.00168.x 17214841 PMC2034354

[B39] Nedergaard J. Ricquier D. Kozak L. P. (2005). Uncoupling proteins: current status and therapeutic prospects. EMBO Rep. 6 (10), 917–921. 10.1038/sj.embor.7400532 16179945 PMC1369193

[B40] Nedergaard J. Cannon B. (2010). The changed metabolic world with human brown adipose tissue: therapeutic visions. Cell Metab. 11 (4), 268–272. 10.1016/j.cmet.2010.03.007 20374959

[B41] Okada-Iwabu M. Yamauchi T. Iwabu M. Honma T. Hamagami K. Matsuda K. (2013). A small-molecule AdipoR agonist for type 2 diabetes and short life in obesity. Nature 503 (7477), 493–499. 10.1038/nature12656 24172895

[B42] Orlandi M. Graziani F. D’Aiuto F. (2020). Periodontal therapy and cardiovascular risk. Periodontol. 2000 83 (1), 107–124. 10.1111/prd.12299 32385887

[B43] Ozcan U. Cao Q. Yilmaz E. Lee A. H. Iwakoshi N. N. Ozdelen E. (2004). Endoplasmic reticulum stress links obesity, insulin action, and type 2 diabetes. Science 306 (5695), 457–461. 10.1126/science.1103160 15486293

[B44] Pihlstrom B. L. Michalowicz B. S. Johnson N. W. (2005). Periodontal diseases. Lancet 366 (9499), 1809–1820. 10.1016/s0140-6736(05)67728-8 16298220

[B45] Polak D. Sanui T. Nishimura F. Shapira L. (2020). Diabetes as a risk factor for periodontal disease—plausible mechanisms. Periodontol. 2000 83 (1), 46–58. 10.1111/prd.12298 32385872

[B46] Puri V. Ranjit S. Konda S. Nicoloro S. M. C. Straubhaar J. Chawla A. (2008). Cidea is associated with lipid droplets and insulin sensitivity in humans. Proc. Natl. Acad. Sci. 105 (22), 7833–7838. 10.1073/pnas.0802063105 18509062 PMC2409392

[B47] Qing H. Desrouleaux R. Israni-Winger K. Mineur Y. S. Fogelman N. Zhang C. (2020). Origin and Function of Stress-Induced IL-6 in Murine Models. Cell 182 (2), 372–387.e314. 10.1016/j.cell.2020.05.054 32610084 PMC7384974

[B48] Ritchie M. E. Phipson B. Wu D. Hu Y. Law C. W. Shi W. (2015). limma powers differential expression analyses for RNA-sequencing and microarray studies. Nucleic Acids Res. 43 (7), e47. 10.1093/nar/gkv007 25605792 PMC4402510

[B49] Saito T. Shimazaki Y. Sakamoto M. (1998). Obesity and periodontitis. N Engl. J. Med. 339 (7), 482–483. 10.1056/nejm199808133390717 9705695

[B50] Saito M. Okamatsu-Ogura Y. Matsushita M. Watanabe K. Yoneshiro T. Nio-Kobayashi J. (2009). High incidence of metabolically active brown adipose tissue in healthy adult humans: effects of cold exposure and adiposity. Diabetes 58 (7), 1526–1531. 10.2337/db09-0530 19401428 PMC2699872

[B51] Samad F. Yamamoto K. Pandey M. Loskutoff D. J. (1997). Elevated expression of transforming growth factor-beta in adipose tissue from obese mice. Mol. Med. 3 (1), 37–48. 10.1007/BF03401666 9132278 PMC2230108

[B52] Samad F. Uysal K. T. Wiesbrock S. M. Pandey M. Hotamisligil G. S. Loskutoff D. J. (1999). Tumor necrosis factor alpha is a key component in the obesity-linked elevation of plasminogen activator inhibitor 1. Proc. Natl. Acad. Sci. U.S.A. 96 (12), 6902–6907. 10.1073/pnas.96.12.6902 10359811 PMC22014

[B53] Sasaki N. Katagiri S. Komazaki R. Watanabe K. Maekawa S. Shiba T. (2018). Endotoxemia by Porphyromonas gingivalis Injection Aggravates Non-alcoholic Fatty Liver Disease, Disrupts Glucose/Lipid Metabolism, and Alters Gut Microbiota in Mice. Front. Microbiol. 9, 2470. 10.3389/fmicb.2018.02470 30405551 PMC6207869

[B54] Schenkein H. A. Papapanou P. N. Genco R. Sanz M. (2020). Mechanisms underlying the association between periodontitis and atherosclerotic disease. Periodontol. 2000 83 (1), 90–106. 10.1111/prd.12304 32385879

[B55] Shibata H. Pérusse F. Vallerand A. Bukowiecki L. J. (1989). Cold exposure reverses inhibitory effects of fasting on peripheral glucose uptake in rats. Am. J. Physiol. 257 (1 Pt 2), R96–101. 10.1152/ajpregu.1989.257.1.R96 2665523

[B56] Stanford K. I. Middelbeek R. J. Townsend K. L. An D. Nygaard E. B. Hitchcox K. M. (2013). Brown adipose tissue regulates glucose homeostasis and insulin sensitivity. J. Clin. Invest. 123 (1), 215–223. 10.1172/jci62308 23221344 PMC3533266

[B57] Subramanian A. Tamayo P. Mootha V. K. Mukherjee S. Ebert B. L. Gillette M. A. (2005). Gene set enrichment analysis: a knowledge-based approach for interpreting genome-wide expression profiles. Proc. Natl. Acad. Sci. U. S. A. 102 (43), 15545–15550. 10.1073/pnas.0506580102 16199517 PMC1239896

[B58] Taniguchi C. M. Emanuelli B. Kahn C. R. (2006). Critical nodes in signalling pathways: insights into insulin action. Nat. Rev. Mol. Cell Biol. 7 (2), 85–96. 10.1038/nrm1837 16493415

[B59] Tonetti M. S. D’Aiuto F. Nibali L. Donald A. Storry C. Parkar M. (2007). Treatment of Periodontitis and Endothelial Function. New Engl. J. Med. 356 (9), 911–920. 10.1056/nejmoa063186 17329698

[B60] Udagawa S. Katagiri S. Maekawa S. Takeuchi Y. Komazaki R. Ohtsu A. (2018). Effect of Porphyromonas gingivalis infection in the placenta and umbilical cord in pregnant mice with low birth weight. Acta Odontol. Scand. 76 (6), 433–441. 10.1080/00016357.2018.1426876 29334319

[B61] van der Lans A. A. Hoeks J. Brans B. Vijgen G. H. Visser M. G. Vosselman M. J. (2013). Cold acclimation recruits human brown fat and increases nonshivering thermogenesis. J. Clin. Invest. 123 (8), 3395–3403. 10.1172/jci68993 23867626 PMC3726172

[B62] Villarroya F. Cereijo R. Villarroya J. Giralt M. (2017). Brown adipose tissue as a secretory organ. Nat. Rev. Endocrinol. 13 (1), 26–35. 10.1038/nrendo.2016.136 27616452

[B63] Villarroya F. Cereijo R. Gavaldà-Navarro A. Villarroya J. Giralt M. (2018). Inflammation of brown/beige adipose tissues in obesity and metabolic disease. J. Internal Med. 284 (5), 492–504. 10.1111/joim.12803 29923291

[B64] Wang J. Duncan D. Shi Z. Zhang B. (2013). WEB-based GEne SeT AnaLysis Toolkit (WebGestalt): update 2013. Nucleic Acids Res. 41, W77–W83. 10.1093/nar/gkt439 23703215 PMC3692109

[B65] Wang G. X. Zhao X. Y. Meng Z. X. Kern M. Dietrich A. Chen Z. (2014). The brown fat-enriched secreted factor Nrg4 preserves metabolic homeostasis through attenuation of hepatic lipogenesis. Nat. Med. 20 (12), 1436–1443. 10.1038/nm.3713 25401691 PMC4257907

[B66] Wang W. Seale P. (2016). Control of brown and beige fat development. Nat. Rev. Mol. Cell Biol. 17 (11), 691–702. 10.1038/nrm.2016.96 27552974 PMC5627770

[B67] Watanabe K. Katagiri S. Takahashi H. Sasaki N. Maekawa S. Komazaki R. (2020). Porphyromonas gingivalis impairs glucose uptake in skeletal muscle associated with altering gut microbiota. FASEB J. 10.1096/fj.202001158R 33197074

[B68] Whitlock G. Lewington S. Sherliker P. Clarke R. Emberson J. Halsey J. (2009). Body-mass index and cause-specific mortality in 900 000 adults: collaborative analyses of 57 prospective studies. Lancet 373 (9669), 1083–1096. 10.1016/s0140-6736(09)60318-4 19299006 PMC2662372

[B69] Yadav H. Quijano C. Kamaraju A. K. Gavrilova O. Malek R. Chen W. (2011). Protection from obesity and diabetes by blockade of TGF-β/Smad3 signaling. Cell Metab. 14 (1), 67–79. 10.1016/j.cmet.2011.04.013 21723505 PMC3169298

[B70] Yamauchi T. Kamon J. Waki H. Terauchi Y. Kubota N. Hara K. (2001). The fat-derived hormone adiponectin reverses insulin resistance associated with both lipoatrophy and obesity. Nat. Med. 7 (8), 941–946. 10.1038/90984 11479627

[B71] Yoneda M. Naka S. Nakano K. Wada K. Endo H. Mawatari H. (2012). Involvement of a periodontal pathogen, Porphyromonas gingivalis on the pathogenesis of non-alcoholic fatty liver disease. BMC Gastroenterol. 12, 16. 10.1186/1471-230x-12-16 22340817 PMC3305584

[B72] Yoneshiro T. Aita S. Matsushita M. Kayahara T. Kameya T. Kawai Y. (2013). Recruited brown adipose tissue as an antiobesity agent in humans. J. Clin. Invest. 123 (8), 3404–3408. 10.1172/jci67803 23867622 PMC3726164

[B73] Yoon M. J. Lee G. Y. Chung J. J. Ahn Y. H. Hong S. H. Kim J. B. (2006). Adiponectin increases fatty acid oxidation in skeletal muscle cells by sequential activation of AMP-activated protein kinase, p38 mitogen-activated protein kinase, and peroxisome proliferator-activated receptor alpha. Diabetes 55 (9), 2562–2570. 10.2337/db05-1322 16936205

[B74] Yu B. L. Zhao S. P. Hu J. R. (2010). Cholesterol imbalance in adipocytes: a possible mechanism of adipocytes dysfunction in obesity. Obes. Rev. 11 (8), 560–567. 10.1111/j.1467-789X.2009.00699.x 20025694

