# Supplementary material for: Endotoxemia by Porphyromonas gingivalis Alters Endocrine Functions in Brown Adipose Tissue
Source: Front Cell Infect Microbiol. 2021 Jan 19;10:580577. doi: 10.3389/fcimb.2020.580577 (PMC7850987; doi:10.3389/fcimb.2020.580577)
Supplement: Supplementary file 1 [file DataSheet_1.pdf]

**Supplementary Table S1. Primers used for Quantitative reverse-transcription PCR analysis.**

| *Gene* | Primers | |
|---|---|---|
| | Sense (5’-3’) | Anti-sence (5’-3’) |
| *Adipoq* | GTTCTACTGCAACATTCCGG | TACACCTGGAGCCAGACTTG |
| *Ccl2* | TTAAAAACCTGGATCGGAACCAA | GCATTAGCTTCAGATTTACGGGT |
| *Cers3* | TTCAAGCATTCCACAAGCAAAC | CAACCTGGCGCTCTGTCAA |
| *Cidea* | TGACATTCATGGGATTGCAGAC | GGCCAGTTGTGATGACTAAGAC |
| *Cxcl14* | GAAGATGGTTATCGTCACCACC | CGTTCCAGGCATTGTACCACT |
| *Fads3* | TGACCTACCAGGCGACAAGT | CAATCAACAGGGGTTTCAGGAA |
| *Fasn* | GGAGGTGGTGATAGCCGGTAT | TGGGTAATCCATAGAGCCCAG |
| *Fgf21* | CTGCTGGGGGTCTACCAAG | CTGCGCCTACCACTGTTCC |
| *Il1b* | GCAACTGTTCCTGAACTCAACT | ATCTTTTGGGGTCCGTCAACT |
| *Il6* | TAGTCCTTCCTACCCCAATTTCC | TTGGTCCTTAGCCACTCCTTC |
| *Lipe* | CCAGCCTGAGGGCTTACTG | CTCCATTGACTGTGACATCTCG |
| *Nrg4* | CCAGCCCATTCTGTAGGTGC | CGCGATGGTAAGAGTGAGGA |
| *Pnpla2* | GGATGGCGGCATTTCAGACA | CAAAGGGTTGGGTTGGTTCAG |
| *Pparg* | CCAGAGTCTGCTGATCTGCG | CCAGAGTCTGCTGATCTGCG |
| *Tnfa* | ACGGCATGGATCTCAAAGAC | AGATAGCAAATCGGCTGACG |
| *Ucp1* | AGGCTTCCAGTACCATTAGGT | CTGAGTGAGGCAAAGCTGATTT |
| *Rn18s* | GTAACCCGTTGAACCCCATT | CCATCCAATCGGTAGTAGCG |
